# Proteomic profiling of urine for the detection of colon cancer

**DOI:** 10.1186/1477-5956-6-19

**Published:** 2008-06-16

**Authors:** Douglas G Ward, Stephen Nyangoma, Howard Joy, Emma Hamilton, Wenbin Wei, Chris Tselepis, Neil Steven, Michael JO Wakelam, Philip J Johnson, Tariq Ismail, Ashley Martin

**Affiliations:** 1 Cancer Research UK Institute for Cancer Studies, University of Birmingham, Edgbaston, Birmingham, B15 2TT, UK; 2 Birmingham Bowel Clinic, University Hospital Birmingham, Birmingham, UK

## Abstract

**Background:**

Colorectal cancer is the second most common cause of cancer related death in the developed world. To date, no blood or stool biomarkers with both high sensitivity and specificity for potentially curable early stage disease have been validated for clinical use. SELDI and MALDI profiling are being used increasingly to search for biomarkers in both blood and urine. Both techniques provide information predominantly on the low molecular weight proteome (<15 kDa). There have been several reports that colorectal cancer is associated with changes in the serum proteome that are detectable by SELDI and we hypothesised that proteomic changes would also be detectable in urine.

**Results:**

We collected urine from 67 patients with colorectal cancer and 72 non-cancer control subjects, diluted to a constant protein concentration and generated MALDI and SELDI spectra. The intensities of 19 peaks differed significantly between cancer and non-cancer patients by both t-tests and after adjusting for confounders using multiple linear regressions. Logistic regression classifiers based on peak intensities identified colorectal cancer with up to 78% sensitivity at 87% specificity. We identified and independently quantified 3 of the discriminatory peaks using synthetic stable isotope peptides (an 1885 Da fragment of fibrinogen and hepcidin-20) or ELISA (β2-microglobulin).

**Conclusion:**

Changes in the urine proteome may aid in the early detection of colorectal cancer.

## Background

Colorectal cancer is the third most common cancer in the developed world and the second most common cause of cancer-related death. The prognosis is clearly related to the stage at which the disease is detected and this observation has led to screening programmes using the faecal occult blood test that have resulted in a significant reduction in mortality [1]. Other stool-based approaches, such as DNA testing [2] are showing promise but blood tests, such as carcinoembryonic antigen (CEA) have been disappointing due to their low sensitivity in patients with early disease, the target population in screening programmes. The application of other serum biomarkers such as MMP-9, complement C3a des-arg and α-defensins [3-5] or proteomic approaches that seek characteristic diagnostic signatures [6] have met with limited success but have shown that, in principle, they are capable of generating sensitivities and specificities that are superior to CEA.

Surface Enhanced Laser Desorption/Ionisation time-of-flight mass spectrometry (SELDI) has been widely applied to serum and plasma in attempts to discover changes in the proteome diagnostic for human cancers (reviewed in [7-9]). This methodology uses on-chip retentate chromatography followed by Matrix Assisted Laser Desorption/Ionisation (MALDI) time-of-flight mass spectrometry to generate spectra or 'proteomic profiles' of biological fluids. The SELDI 'ProteinChip Arrays' used in profiling studies are typically immobilised metal ion (IMAC) or ion exchange surfaces. A more flexible approach is to pre-fractionate or de-salt using IMAC, reverse phase or ion exchange beads (e.g. ref [10]). The intensities of the peaks in the resulting mass spectra can be compared between patient cohorts and changes associated with disease state used to build diagnostic models. Early studies using this technology attempted to achieve discrimination by identifying 'signatures' characteristic of specific disease groups (e.g. [11]) whereas more recent studies have attempted to identify the polypeptides to which these peaks correspond to enable immunoassays to be developed. It is important that experiments are well designed and executed to avoid any systematic bias between the cohorts being compared other than specific disease status [7,12]. Our group [6] and others [13-17] have used SELDI and MALDI to show that colorectal cancer causes detectable changes in the serum proteome. Some of the peaks with altered intensities in colorectal cancer patients have been identified as α-defensins, apolipoprotein C1, complement C3a des-arg, α1-antitrypsin and transferrin [6,13].

Tumour-related proteolytic activity, associated with tissue invasion and migration [18,19], might result in disease specific patterns of proteolytic fragments and these, being small (<15 kDa) may be detectable by mass spectrometry. The proteolytic fragments of interest are likely however to be present at very low concentrations and effectively masked by abundant serum proteins (albumin, immunoglobulins, etc). We therefore decided to seek such fragments in urine, effectively a natural ultra-filtrate of serum with the large abundant serum proteins excluded. SELDI studies on urine have shown that good quality spectra can be obtained from urine and that renal, bladder and prostate cancers produce proteomic changes [20-26]. More recently, Ye *et al *reported proteomic changes in the urine of ovarian cancer patients [27].

This report is a first attempt to detect colorectal cancer associated changes in urine by proteomic profiling. We collected urine from 67 colorectal cancer patients and 72 non-cancer controls. The cancer patient group consisted of 40 individuals with 'early disease' (Dukes stage A or B i.e. neoplasia confined to the gut wall) and 27 individuals with 'late disease' (Dukes C or D i.e. lymph node involvement or distant metastases). Samples were normalised with respect to protein concentration and, following trials of different MALDI and SELDI methods using a pooled sample, were assayed by MALDI with/without de-salting and Cu^2+ ^loaded IMAC ProteinChip Arrays. We find a number of proteomic changes that may have utility in the diagnosis of colorectal cancer.

## Methods

### Patient Information

Ethical approval for this study was granted by the South Birmingham LREC (05/Q2702/17). All subjects gave informed consent, were fasted overnight and midstream urine was then collected between 9 am and midday. Samples were kept on ice and transferred to the laboratory within 5 hours, centrifuged at 3000 rpm for 20 min, aliquotted and stored at -80°C. Individuals below 40 or above 90 years old were excluded from the study as were samples with a protein concentration of <20 μg protein per ml (14 individuals) or >500 μg protein per ml (5 individuals) as assessed by the bicinchonninic acid protein assay (Pierce). After exclusions we retained 139 samples (72 controls and 67 patients, Table 1).

**Table 1:** Patient Information.

	No. Subjects	Mean age (SD)	Mean [protein] (μg/ml) (SD)	Male/Female	Stage (Early/Late)
Non-cancer controls	72	66.3 (11.5)	90 (66)	36/36	na
Colorectal cancer patients	67	74.2 (9.0)	108 (79)	45/22	40/27

Table 1 shows the number of individuals in each group, their gender and the mean age and urine protein concentration and cancer stage (early = Dukes' A & B, late = Dukes' C & D). Patients whose urine was <20 μg protein per ml (14 individuals) or >500 μg protein per ml (5 individuals) have been excluded.

### Proteomic Profiling

Prior to profiling urine samples were diluted to 20 μg protein/ml with deionised water. SELDI and MALDI spectra were acquired on a PBSIIc ProteinChip reader calibrated with ACTH, insulin, ubiquitin, cytochrome C, myoglobin and albumin.

MALDI spectra of neat urine were obtained by applying 1 μl of 20 μg/ml sample to 3 random positions on GoldChips followed by air drying and overlaying with 1 μl of saturated sinapinic acid in 50% acetonitrile/49.5% water/0.5% TFA. Data were collected up to m/z 20,000 and spectra represent the average of 480 laser shots. MALDI spectra were also obtained following de-salting on ClinProt C8 magnetic beads (BrukerDaltronic) by mixing urine (80 μl of 20 μg/ml) with 20 μl of 5% trifluoroacetic acid containing 2.5 μl of C8 beads and shaking for 5 min. Following two washes with 0.1% TFA the bound material was eluted with 15 μl of saturated sinapinic acid in 50% acetonitrile/49.5% water/0.5% TFA and 1 μl applied to 3 random positions on GoldChips.

SELDI spectra were obtained using Cu^2+ ^loaded IMAC30 ProteinChip arrays and a 96-well bioprocessor as described previously [28]. The 20 μg/ml samples were mixed 1:1 (1 M NaCl, 200 mM sodium phosphate (pH 7.0)) and 100 μl of this mix added to each well in the bioprocessor. Binding was allowed to proceed for 30 min at room temperature with shaking at 900 rpm. The ProteinChip arrays were then washed 4 times using 200 μl of 500 mM NaCl, 100 mM sodium phosphate (pH 7.0) (5 min with shaking) followed by a water rinse. The chips were allowed to dry and 2 × 1 μl of a 50% saturated solution of sinapinic acid in 50% acetonitrile/49.5% water/0.5% TFA applied to each spot. Data were collected over m/z 0–20,000 and 0–200,000 ranges (480 laser shot each). All samples were processed once in random order and then a second time, again in random order.

### Data Analysis

Spectra were normalised to total ion current and baselines subtracted using Ciphergen ProteinChip software. Peaks were detected and clustered using Biomarker Wizard software (default settings except for a requirement for a peak to be detected in more than 5% of the samples). Pair wise correlation coefficients were calculated for the peak intensities of experimental replicates. The MALDI spectra were obtained in triplicate and the replicate with the poorest correlation with the other two replicates was discarded. The peak intensities for the remaining experimental replicates were then averaged prior to further analysis. Samples which did not produce 2 spectra with a regression coefficient higher than 0.94 (MALDI) or 0.84 (SELDI) were excluded from the analyses. Following this QC procedure the number of individuals in each dataset was: MALDI: 123 (55 cancer), MALDI-DS: 119 (53 cancer), SELDI: 116 (55 cancer), with 105 individuals represented in all 3 datasets. Two-sample t-tests and multiple linear regression models (LM) were used to identify cancer associated changes in peak intensities using log (base 2) transformed data (the t-tests test for a difference in means of the 2 groups whereas the LM tests for an association with cancer rather than age, gender, protein concentration or sample collector). For the peaks that showed significance in both statistical tests the multiple fractional polynomials method (MFP) [29] was used to determine optimal (linear/non-linear) relationships between peak intensities and the presence or absence of colorectal cancer and the combinations of transformed peaks that lead to improvements in discrimination. The transformed data was then used for class prediction by logistic regression (LR). Leave-one-out cross-validation was used to estimate classification errors. LM, MFP and LR were carried out using the *lm, mfp *and *glm *objects of R statistical package [29].

### Identification of putative biomarkers

The polypeptides underlying the peaks of interest were purified and identified by tandem mass spectrometry. The purifications were monitored at each step by MALDI or SELDI. The initial step in the purifications was to harvest peptides and proteins from 10–50 ml of urine using C18 SPE cartridges (Phenomenex). Trifluoroacetic acid (TFA) was added to the urine to a concentration of 0.5% v/v prior to binding and proteins eluted sequentially with 10, 20, 30 and 40% v/v acetonitrile in water/0.1%TFA. This was followed by anion exchange fractionation as described previously [6]. Briefly, the sample was dissolved in 50 mM Tris/HCl (pH 9) and applied to Q Ceramic HyperD F anion exchange resin (Pall). Proteins were eluted sequentially at pH 7, 5, 4 and 3. This was followed by reverse phase HPLC (4.6 by 150 mm C18, Phenomenex)) in 0.1% TFA and proteins eluted using an acetonitrile gradients tailored for each purification (based on the SPE). Polypeptides <3 kDa were analysed directly by liquid chromatography tandem mass spectrometry (LC-MS/MS). Polypeptides >3 kDa were subjected to SDS-PAGE (12% NuPAGE, MES running buffer, Invitrogen) and the bands with appropriate mobility excised and digested with trypsin prior to LC-MS/MS) [28]. We used a ThermoFinnigan LCQ Deca XP Plus Ion-Trap linked directly to an LC Packings/Dionex Ultimate nanobore HPLC system to identify peptides by LC-MS/MS. Data was filtered using Xcorr values of 1.5, 2 and 2.5 for singly, doubly and triply charged parent ions respectively and only first hits were considered. MS/MS data was searched against the IPI human database (version 3.36) using SEQUEST. Further evidence for the identifications was obtained by accurate mass determinations of peptides (ProTOF 2000 orthogonal time-of-flight MALDI mass spectrometer) or using an immuno-MS approach as previously described [6,28,30].

## Results

### Establishing Profiling Conditions

**Figure 1:**
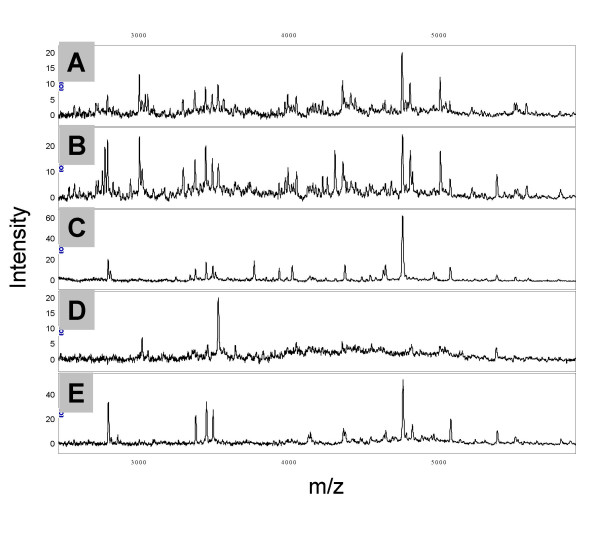
MALDI and SELDI spectra. Spectra obtained from a pooled urine sample. A: MALDI without desalting, B: MALDI with desalting, C: CM10 ProteinChip array, D: Q10 ProteinChip array, E: Cu^2+ ^loaded IMAC30 ProteinChip array.

A pooled urine sample (containing urine from 5 individuals) was analysed by MALDI with/without desalting and by SELDI on 3 ProteinChip array surfaces. The resulting spectra are shown in Figure 1. The MALDI spectra without de-salting contained 48 peaks in the m/z 1,500–20,000 range with a signal to noise ratio greater than 5. De-salting increased this to 63 peaks. Q10 and CM10 ProteinChip Arrays were prepared at pH 7 and produced 46 and 53 peaks respectively. The IMAC chip produced 67 peaks between m/z 1500 and 20,000. In this preliminary work MALDI spectra were acquired up to m/z 200,000, however, they contained very little information above m/z 15,000 whereas the SELDI spectra contained a number of peaks in the m/z 20,000–200,000 range.

### MALDI Profiling Results

MALDI spectra were acquired in triplicate on diluted urine samples (20 μg protein/ml). Following total ion current normalisation, 101 peaks with a signal to noise ratio greater than 5 were detected between m/z 1500 and 20,000. The mean coefficient of variation (CV) of peak intensity for peaks with intensity >5 and <30 in the QC sample was 22% (a pooled urine sample analysed at 30 random positions during the experiment). We tested for significant differences in peak intensity between the non-cancer controls and the colorectal cancer patients using two-sample t-tests and multiple linear regressions (LM). LM was used to confirm that changes in peak intensity apparently associated with colorectal cancer were not due to the slight imbalances in age, gender or urinary protein concentration in our cancer patient and non-cancer control groups (Table 1). Nine peaks showed up as cancer related (significant) using a combination of both statistical tests (p < 0.05) and are shown in Table 2.

**Table 2:** Cancer associated MALDI/SELDI peaks.

Method	m/z	P value (t-test)	P value (LM)	Fold change	AUROC
MALDI	1606*	0.0001	0.0006	0.72	0.71
MALDI	2051*	0.0253	0.0338	1.20	0.64
MALDI	2195*	0.0027	0.0373	0.67	0.65
MALDI	2254*	0.0023	0.0030	1.24	0.69
MALDI	4169*	0.0074	0.0083	1.25	0.65
MALDI	5011	0.0068	0.0009	1.23	0.68
MALDI	5239*	0.0009	0.0002	1.31	0.68
MALDI	11025*	0.0186	0.0430	0.48	0.60
MALDI	11400*	0.0146	0.0133	0.44	0.59
MALDI-DS	2193*	0.0001	0.0001	0.53	0.70
MALDI-DS	2758*	0.0029	0.0029	0.74	0.64
MALDI-DS	4758*	0.0121	0.0408	0.70	0.64
MALDI-DS	5812*	0.0052	0.0070	0.59	0.63
MALDI-DS	6250	0.0426	0.0108	1.22	0.60
SELDI	1885	0.0153	0.0450	1.28	0.64
SELDI	6086*	0.0259	0.0050	1.14	0.61
SELDI	11750*	0.0019	0.0032	1.67	0.67
SELDI	11960*	0.0005	0.0182	1.65	0.68
SELDI-HR	39480	0.0217	0.043	0.94	0.62
SELDI-HR	53760*	0.0293	0.0150	0.98	0.64

Table 2 shows the m/z ratios, p-values obtained by t-test and multiple linear regression (LM), fold-change (cancer mean intensity/control mean intensity) and area under the receiver operator characteristic curve (AUROC) for all peaks which obtained p < 0.05 in both statistical tests. The peaks that are significant in one or both statistical tests if only the early stage cancers are compared to the non-cancer controls are marked with an asterisk.

MALDI spectra were also acquired in triplicate following concentration and de-salting on C8 magnetic beads (MALDI-DS). We detected 97 peaks with a signal to noise ratio greater than 5 between m/z 1500 and 20,000. The mean CV of peak intensity for peaks with intensity >5 and <30 in the QC sample spectra was 20%. Five peaks attained significance in both t-tests and LM (Table 2). The most significant peak at m/z 2193 is, on average, halved in intensity in the cancer patients and was also significantly decreased in the data obtained without de-salting.

### SELDI Profiling Results

Duplicate spectra were acquired for each sample over m/z 1500–20,000 and 20,000–200,000 ranges. One hundred peaks were detected in the low mass range and a further 35 peaks were detected in the high mass range. The mean CV of peak intensity for peaks with intensity >5 and <30 in the QC sample spectra was 29%. The intensities of 6 peaks were significantly associated with cancer patients according to t-tests and LM (Table 2).

### Comparison of MALDI and SELDI profiling

**Figure 2:**
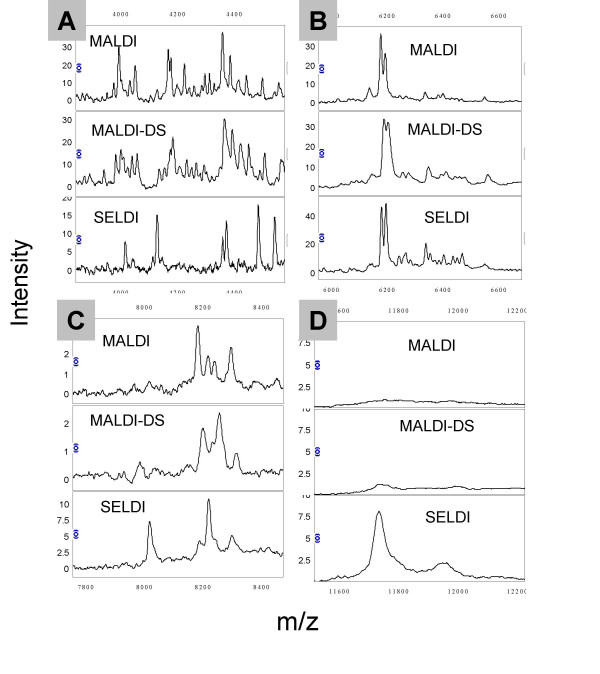
Comparison of MALDI and SELDI urine profiles. Figures 2 a-d show expanded regions of the MALDI, MALDI-DS and SELDI spectra from one female non-cancer subject.

**Figure 3:**
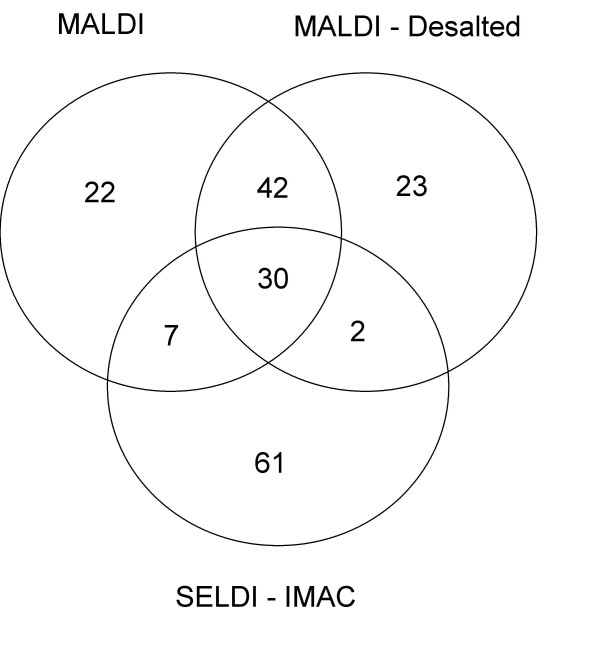
Overlap between SELDI and MALDI datasets. This Venn diagram shows the number of peaks detected in urine between m/z 1500 and 20,000 by MALDI, MALDI-DS and SELDI and the overlap between the 3 methods.

**Figure 4:**
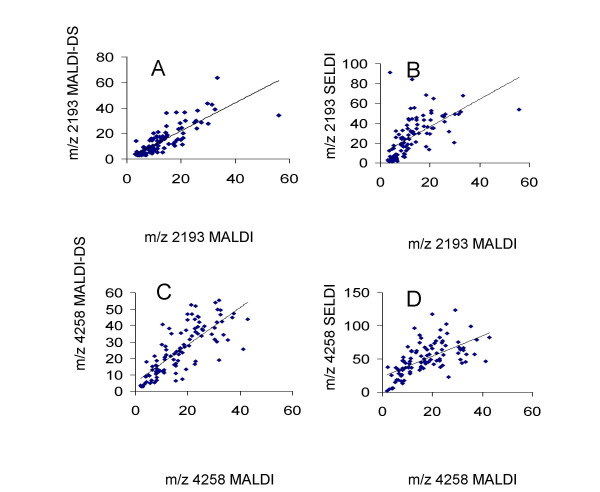
Correlation between peak intensities in different datasets. Figure 4 shows the correlations between peak intensities in the MALDI and MALDI-DS and MALDI and SELDI datasets for 2 peaks: m/z 2193 (A & B) and m/z 4258(C & D).

All 3 profiling methods produced spectra with ~100 peaks between m/z 1500 and 20,000. Figures 2a–d show expanded views of regions of the MALDI, MALDI-DS and SELDI spectra from one urine sample: some peaks are present in both MALDI methods but not SELDI (eg Fig 2a) whereas other peaks are better detected by SELDI than MALDI (e.g. the 11.7 kDa peak of β2-microglobulin in Fig 2d). The redundancy between the profiling methods can be estimated if one assumes that peaks falling within a m/z window of 0.3% in the 3 datasets represent the same molecular ion (Figure 3). Approximately 72% of peaks are shared between the MALDI and MALDI-DS datasets whereas redundancy between the MALDI datasets and the SELDI is <40%. If a peak is detected in 2 or more datasets then a strong correlation between the intensities in the datasets might be expected and we find this to be the case for many peaks (examples shown in Figure 4). We find a positive correlation between the intensities of all peaks shared between the MALDI datasets with an average correlation coefficient of 0.61 (range 0.2–0.87). The correlations between MALDI and SELDI datasets are not as strong with an average correlation coefficient of 0.44 (range -0.13–0.85) and 18 out of 39 peaks having a correlation coefficient >0.50. For 9 of the 39 shared peaks within a 0.3% m/z window little or no correlation existed (correlation coefficient <0.2).

### Class Prediction Models

**Figure 5:**
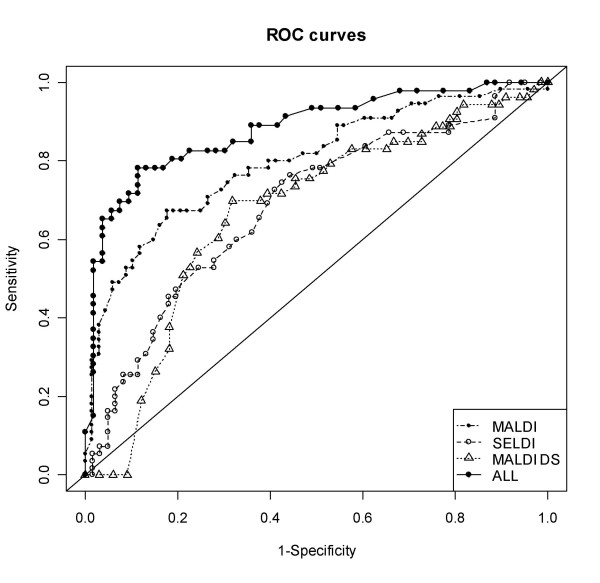
Receiver operator characteristic analysis of classification models. The MALDI model is represented as a dot-dash line with solid symbols, the MALDI-DS model as a dotted line with open triangles, the SELDI model as a dashed line with open circles and the 'ALL' model as a solid line with solid symbols (the diagonal represents no discrimination).

LR was used to discriminate between non-cancer controls and cancer patients based on the peaks in their urine profiles. The variables used in this analysis were the combinations of proteins (or their transformations) selected by MFP from among those found to be cancer related. The sensitivities and specificities estimated by leave-one-out cross-validation are shown in Table 3: all 3 datasets provided some level of discrimination with the MALDI dataset producing the best classifier with 65% sensitivity at 84% specificity. A model built using the significant peaks from all 3 datasets selected peaks with m/z ratios of 1606, 2051 and 5011 (MALDI) and 4758 (MALDI-DS) as discriminators and yielded 78% sensitivity and 87% specificity. The models built on MALDI, MALDI-DS, SELDI datasets and a combination of these datasets correctly detected 65%, 63%, 48% and 70% of the early stage cancers (Duke's stages A & B) indicating that, as suggested by our statistical analysis (Table 2), the proteomic changes are not confined to late stage disease. ROC curves for the models built on the 3 datasets are shown in Figure 5.

**Table 3:** Class Prediction Model Results.

Dataset	Sensitivity	Specificity	Misclassification Rate	AUROC
MALDI	65 (52–78)	84 (75–93)	24	0.80
MALDI-DS	68 (55–80)	68 (57–79)	32	0.67
SELDI	51 (38–64)	75 (64–86)	34	0.69
ALL	78 (66–90)	87 (78–96)	17	0.88

Table 3 summarises the logistic regression models tested by leave-one-out cross-validation. Sensitivity, specificity and misclassification rate are presented as percentages (95% confidence intervals). The m/z values of the peaks used in the logistic regressions are, MALDI: 1606, 2254, 5011, MALDI-DS: 2193, SELDI: 1885 and 11960, ALL: 1606, 2251, 4758, 5011. Number of samples in each dataset: MALDI: 123 (55 cancer), MALDI-DS: 119 (53 cancer), SELDI: 116 (55 cancer).

### Polypeptide Identifications

**Figure 6:**
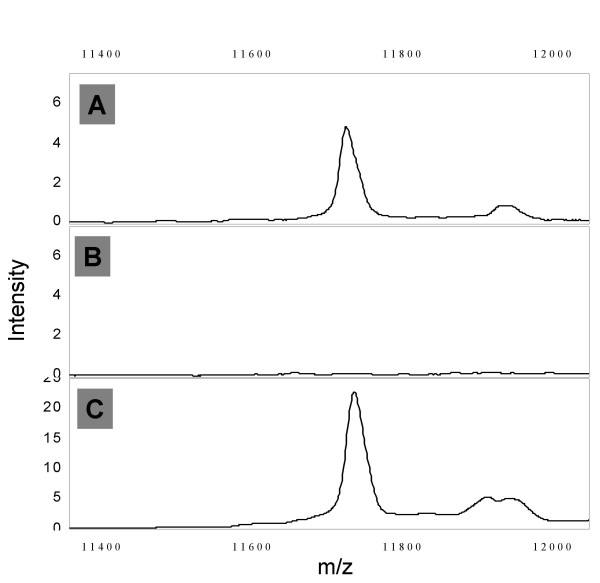
β2-microglobulin immuno-MS. Panel A shows the IMAC-SELDI spectrum of neat serum, panel B the spectrum of the same serum following depletion with protein G beads loaded with rabbit polyclonal anti-serum to human β2-microglobulin (Sigma M8523) and panel C the spectrum of the eluted β2-microglobulin.

**Figure 7:**
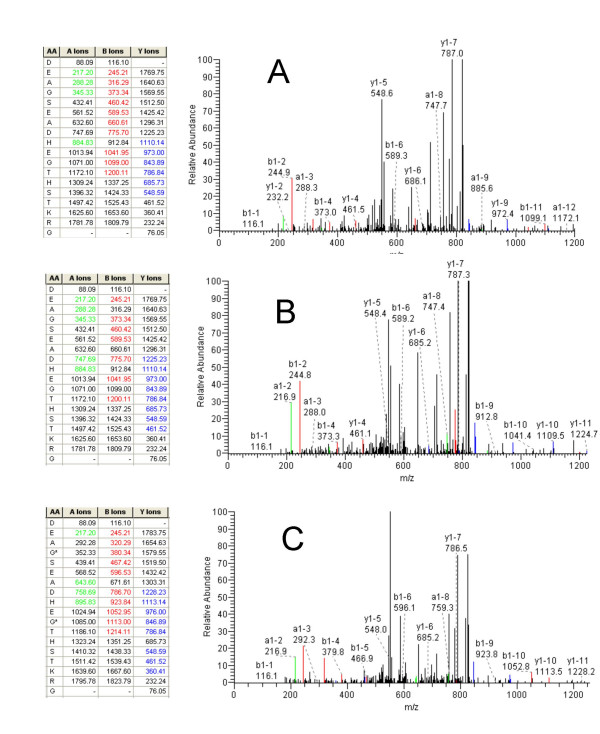
Fibrinogen peptide MS/MS data. Figure 7a shows one of the MS/MS spectra used to identify the m/z 1885 SELDI peak as the fragment of fibrinogen DEAGSEADHEGTHSTKRG (3+ parent ion, 1+ fragments). Figure 7b shows MS/MS analysis of synthetic DEAGSEADHEGTHSTKRG. Figure 7c shows MS/MS analysis of synthetic DEAGSEADHEGTHSTKRG with ^13^C + ^15^N labelled alanine at positions 3 and 7 and glycine at positions 4 and 11. All spectra obtained by LC-MS/MS as described in the Methods section.

We have determined the identity of 4 of the peaks most significantly associated with colorectal cancer. The most significantly cancer associated peak in the MALDI-DS experiment with a m/z ratio of 2193 is decreased in intensity in the cancer patients and is also significantly decreased in the MALDI experiment (m/z 2195, Table 2). This is a prominent peak in urine spectra whose identity has previously been described in the literature as hepcidin-20 (along with hepcidin-22 and hepcidin-25 at m/z 2433 and 2788) [31]. We gained strong support for the m/z 2193 peak arising from hepcidin-20 by obtaining an accurate m/z value for the monoisotopic H^+ ^ion underlying this peak using an orthogonal time-of-flight MALDI mass spectrometer (ProTOF 2000). The accurate m/z value (2190.838) matches the calculated m/z of hepcidin-20 corrected for the 4 internal disulphide bridges in the structure (2190.840). Additionally, the mass of the peak increases by 464.2 Da when treated with DTT and iodoacteamide, again consistent with 4 disulphide bridges (data not shown). We have identified the SELDI peaks elevated in cancer at m/z 11720 and 11920 as β2-microglobulin from 3 tryptic fragments giving 23% sequence coverage (the 11920 peak is probably a sinapinic acid adduct as this is absent when α-cyano-hydroxycinnamic acid is used as the matrix, data not shown). This identification was confirmed using an immuno-MS approach (Figure 6). A second peak elevated in the cancer patients in the SELDI survey at m/z 1885 was identified as a fragment of the α-chain of fibrinogen (DEAGSEADHEGTHSTKRG). This identification is based on LC-ESI-MS/MS of the purified 'peak' without prior tryptic digestion. The same MS/MS data and database match were obtained in several repeat experiments. We have not attempted to confirm this identification by immuno-MS as the 1885 peak represents such a small portion of fibrinogen but have obtained an accurate monoisotopic H^+ ^m/z of 1883.816 (predicted 1883.811) Additionally, a synthetic version of this peptide produced identical MS and MS/MS data (Figure 7).

### Independent quantification of the identified polypeptides

**Figure 8:**
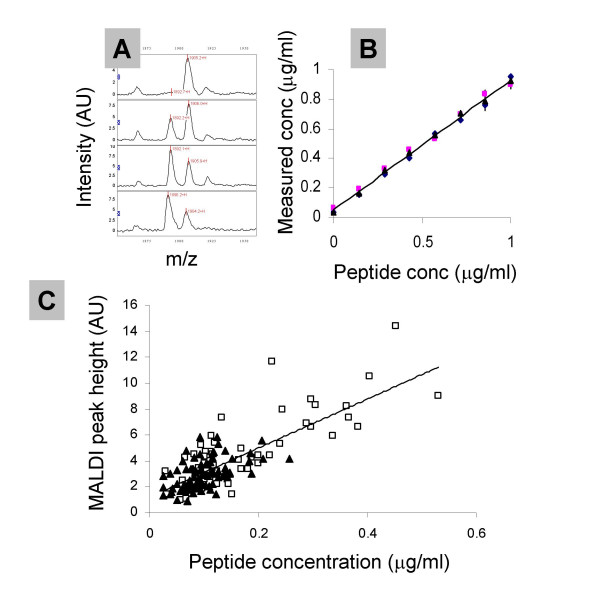
Spiking of stable isotope labelled fibrinogen peptide into urine samples. Panel A shows the MALDI spectra of a urine sample spiked with 0.5 μg/ml heavy peptide and 0, 0.3, 0.7 and 1 μg/ml light peptide. Panel B plots the 'measured' concentration of light peptide against the actual concentration added (3 experiments in different urine samples devoid of the endogenous peptide). Panel C plots MALDI peak height at m/z 1885 for each sample in the study against 'measured' concentration (from the light/heavy peptide peak intensity) with non-cancer controls indicated by solid triangles and cancer patients by open squares.

Synthetic versions of the DEAGSEADHEGTHSTKRG fibrinogen peptide with/without ^13^C/^15^N alanine and glycine residues (average H^+ ^ion m/z values of 1885 and 1899) were synthesised by AltaBioscience, University of Birmingham. Figure 8a shows MALDI spectra of a urine sample devoid of the endogenous peak spiked with 0.5 μg/ml heavy peptide and increasing concentrations of light peptide. The concentration of the light peptide can be calculated from the ratio of the peak heights at m/z 1885 and 1899 and the concentration of heavy peptide in the sample. In the experiment shown in Figure 8b we added 0.5 μg/ml heavy peptide and varying concentrations of light peptide to 3 urine samples low in the endogenous 1885 peak. There is close agreement between the concentration of light peptide and the concentration calculated from the MALDI peak heights. This experiment worked equally well on IMAC chips (data not shown). Figure 8c shows the correlation (r = 0.607) between the height of the 1885 peak in MALDI spectra following total ion current normalisation and the concentration of the DEAGSEADHEGTHSTKRG peptide (calculated from the ratio of the 1885 and 1899 peak heights) in the urine of colorectal cancer patients and non-cancer controls. We find that, as with the peak intensity in the profiles, the concentration of this peptide (as calculated by the labelled peptide spiking approach) is significantly elevated in the colorectal cancer patients at 183 ± 117 ng/ml versus 117 ± 54 ng/ml (mean ± SD, t-test p = 0.0005).

**Figure 9:**
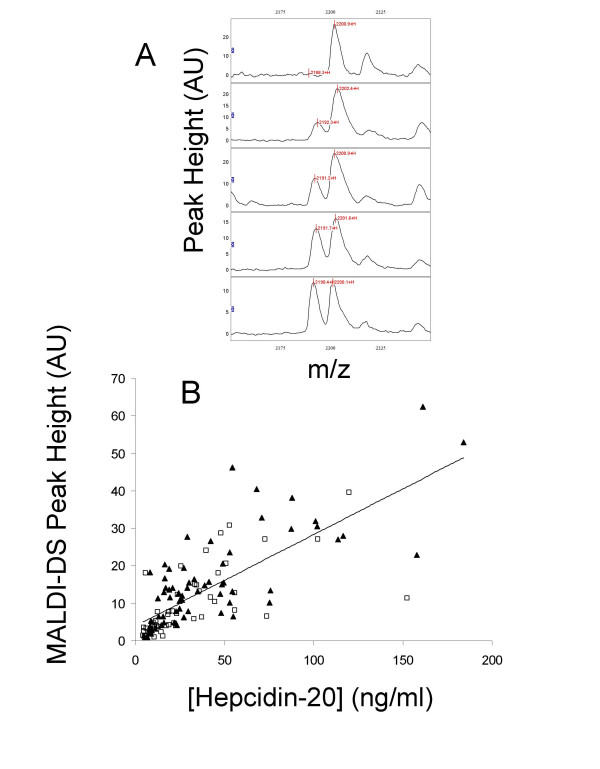
Determination of urinary hepcidin-20 by comparison to stable isotope labelled hepcidin-20. All urine samples were diluted to 20 μg protein/ml and spiked with labelled hepcidin-20 prior to desalting on C8 beads and MALDI mass spectrometry. Figure 9a shows spectra of 5 urines with increasing levels of endogenous hepcidin-20 (m/z 2192) spiked with 80 ng/ml labelled hepcidin-20 (m/z 2202). Figure 9b shows the correlation between the hepcidin-20 concentrations determined from spiked experiments and the peak intensities in the original MALDI-DS experiment. Non-cancer controls are indicated by solid triangles and cancer patients by open squares.

Labelled hepcidin-20 was synthesised with a ^13^C/^15^N phenylalanine residue (Δ mass = 10 Da, giving 2202 Da), incubated overnight at room temperature in 6 M urea, 100 mM ammonium bicarbonate to allow disulphide bridge formation and purified by RP-HPLC. Correct folding/disulphide bridge formation was surmised from an 8 Da reduction in mass and amide proton NMR shifts consistent with published structures (data not shown) [32,33]. The folded labelled hepcidin-20 was then spiked in to urine samples prior to desalting on C8 beads and acquisition of MALDI mass spectra. Hepcidin-20 concentrations were then determined from the ratio of peak intensities at m/z 2192 and m/z 2202. MALDI spectra showing the endogenous hepcidin-20 and labelled hepcidin-20 peaks in 5 urine samples spiked with 80 ng/ml labelled hepcidin-20 are shown in Figure 9a. Figure 9b shows the relationship between the hepcidin-20 peak intensity in the MALDI-DS experiment and the concentration calculated from the m/z 2192/2202 ratio (r = 0.568). The mean hepcidin-20 concentration in the cancer patients is lower than that of the non-cancer controls (30.2 ± 9.1 versus 39.6 ± 12.5 ng/ml), however this is not statistically significant (p = 0.132).

**Figure 10:**
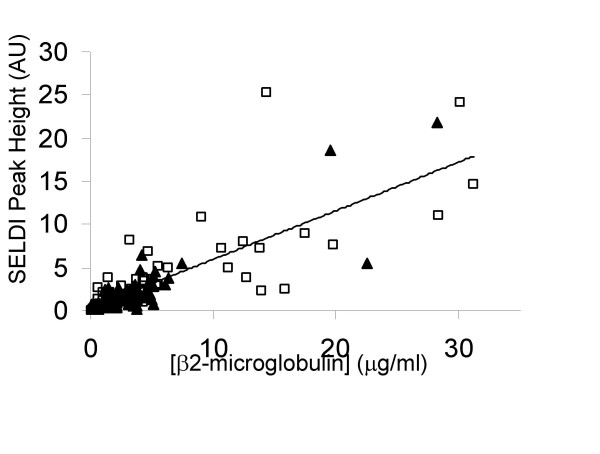
Correlation between SELDI peak intensity at m/z 11750 and β2-microglobulin concentration. The β2-microglobulin was determined in urine samples diluted to 20 μg protein/ml using a sandwich ELISA (Demeditec Diagnostics GmbH, DE-05BM). Non-cancer controls are indicated by solid triangles and cancer patients by open squares.

The relationship between the SELDI peak at m/z 11750 and β2-microgobulin concentration determined by ELISA is shown in Figure 10. There is strong positive correlation (r = 0.656) and the mean concentration in the cancer patients is significantly higher than in the non-cancer controls (5.90 ± 7.08 versus 3.46 ± 4.42 μg/ml, p = 0.018).

## Discussion

We have used three profiling methods to characterise proteomic differences between the urine of colon cancer patients and non-cancer controls. Although no peaks were found to be unique to either the cancer patients or controls a number of changes in peak intensity were significantly associated with colon cancer and these, in conjunction with class prediction models, yielded a diagnostic sensitivity of 78% and specificity of 87%. These values are higher than those obtained with serum CEA but not as good as those obtained in similar profiling studies conducted on serum and the number and significance of proteomic changes in urine also appear to be less than in serum [6,14,15]. Although the reduction in concentration of abundant serum proteins in urine relative to serum should aid detection of more informative lower abundance species, urine is one step further removed from the tumour than blood and may have a more variable composition.

We find that, although the 3 profiling methods used here are complimentary (Figure 3) the simplest method, MALDI, found the most cancer associated proteomic changes and produced the best single method classifier. Both MALDI-DS and SELDI involve a binding step with selectivity for certain peptides which, by chance, excluded some of the polypeptides of interest. The MALDI-DS data gave marginally better reproducibility than MALDI whereas the SELDI data showed the highest variability of the 3 methods (probably partly due to the concentration of protein at the binding step being sub-optimal when using urine for SELDI) possibly masking some effects of cancer on the urinary proteome. When MFP was applied to all of the significant peaks from the 3 methods, peaks from both the MALDI and MALDI-DS datasets were selected to generate a classifier that performed better than that based on the MALDI data alone (Table 3 and Figure 5).

It is challenging to identify all of the peaks of interest in SELDI/MALDI profiling work for a range of reasons: we attempted to identify the discriminatory peaks with m/z ratios of 1606, 2051 and 5011. The m/z 1606 peak proved difficult to purify or obtain MS/MS data on. The m/z 2051 peak gave good MS/MS data but we were unable to obtain a database match and the m/z 5011 peak, although successfully purified, did not appear to generate identifiable tryptic peptides. Furthermore digestion using Asp-N, Glu-C, Lys-C or Arg-C failed to generate identifiable peptides. However we did successfully identify the polypeptides responsible for discriminatory peaks at m/z 1885, 2193 and 11,750. This is a prerequisite for both the development of alternative assay platforms for candidate biomarkers and for understanding the mechanisms underlying these proteomic changes. The cancer associated proteins that we have identified are hepcidin-20, β2-microglobulin and a 18 residue fragment of the α-subunit of fibrinogen. All 3 are proteins primarily synthesised in the liver and are likely to reflect secondary effects of cancer rather than direct secretion/leakage from the tumour itself. Hepcidin-20, decreased in the urine of cancer patients, is an N-terminally truncated form of the hormone hepcidin-25 which is elevated by iron overload and inflammation [34]. Hepcidin-25 which is involved in iron homeostasis (which may be linked to colorectal cancer [35]) is not significantly different in the urine of the cancer patients and non-cancer controls although we did find a positive correlation with T stage [36]. The intensities of the peaks corresponding to β2-microglobulin are, on average, elevated in the urine of the colorectal cancer patients. Serum levels of β2-microglobulin are known to be elevated during infection and in certain lymphoid malignancies [37]. We have previously detected β2-microglobulin as slightly increased in the serum of hepatocellular carcinoma and colorectal cancer patients [6,30]. It is attractive to link the increased urine β2-microglobulin concentration with the elevated serum concentration (although it could also be caused by decrease tubular reabsorbtion). The m/z 1885 peak corresponding to a 18 residue fragment of fibrinogen is present in non-cancer controls, but has an increased intensity in the colorectal cancer patients. Several proteomic profiling studies have now associated proteolytic fragments of abundant serum proteins with malignant disease [38,39]. Thus the increase in the level of fibrinogen fragment, presumably generated in the blood, may arise from increased proteolytic activity or increased total fibrinogen levels (elevated fibrinogen has been reported in the serum of colorectal cancer patients [40]).

As SELDI and MALDI are not quantitative techniques we have used alternative assays to test whether the cancer associated changes in intensity of the MALDI/SELDI peaks of the 3 identified polypeptides truly reflect changes in concentration. Antibodies specific for the 1885 Da fragment of fibrinogen and hepcidin-20 do not exist so we spiked stable isotope labelled versions of these peptides in to samples prior to MALDI mass spectrometry. These internal standards should behave in an identical manner to the endogenous peptides during sample preparation and ionisation and enable peptide concentration determination regardless of inter-sample influences on the mass spectra. β2-microglobulin was determined by ELISA. For all 3 polypeptides a strong positive correlation (r>0.5) was found between MALDI/SELDI peak height and concentration indicating that peak heights can, in many cases, be used as an indicator of relative concentration. For one of the three polypeptides, hepcidin-20, the association with cancer lost statistical significance when the concentration was determined relative to the stable isotope standard. This, perhaps unsurprisingly, suggests that statistically significant changes in proteomic profiling experiments will not always yield useful biomarkers.

Even with effective screening tools such as faecal occult blood testing, that are now entering routine clinical practice, large numbers of 'false-positive' results will be generated, i.e. the faecal occult blood test is of low specificity and the requisite, more specific follow-up examinations such as colonoscopy represent a significant health care burden. If our preliminary figures for sensitivity can be improved it is conceivable that proteomic analysis of urine might, by identifying those with a low probability of having tumours, permit prioritisation of investigation to those at highest risk. What is more, the detectable cancer associated proteomic changes in urine may compliment those detected in serum [6] and a combined analysis might improve both sensitivity and specificity for early disease diagnosis.

## Conclusion

The experiments presented here show that colon cancer causes changes in the urinary proteome that can be used to discriminate between non-cancer controls and patients with early stage colorectal cancer. We find that, although there is considerable overlap between the 2 MALDI methods and the IMAC SELDI data, all 3 profiling methods detect unique peaks. It would be insightful to collect serum and urine from larger cohorts of non-cancer controls and colorectal cancer patients and to analyse the peptidome and proteome in depth to fully assess the diagnostic potential of these proteomic changes.

## Abbreviations

AUROC: area under the ROC curve; CEA: carcinoembryonic antigen; CV: coefficient of variation; IMAC: immobilised metal ion chromatography; LM: multiple linear regression model; LR: logistic regression; MALDI: matrix assisted laser desorption/ionisation; MALDI-DS: MALDI following de-salting on C8 beads; MFP: multiple fractional polynomials; SELDI: surface enhanced laser desorption/ionisation; ROC: receiver operator characteristic.

## Competing interests

The authors declare that they have no competing interests.

## Authors' contributions

DGW designed and executed all experimental work, SN performed statistical analyses, HJ and EH collected samples and collated clinical information, WW advised on statistical analyses, CT advised on hepcidin, NS supervised EH, MJOW supervised HJ, PJJ provided biomarker advice and funding, TI was the clinical lead and supervised HJ and EH, AM conceived the project.
